# Supplementary material for: MEG source imaging detects optogenetically-induced activity in cortical and subcortical networks
Source: Nat Commun. 2021 Sep 6;12:5259. doi: 10.1038/s41467-021-25481-y (PMC8421372; doi:10.1038/s41467-021-25481-y)
Supplement: Supplementary file 1 — Supplementary Information [file 41467_2021_25481_MOESM1_ESM.pdf]

Supplementary Information

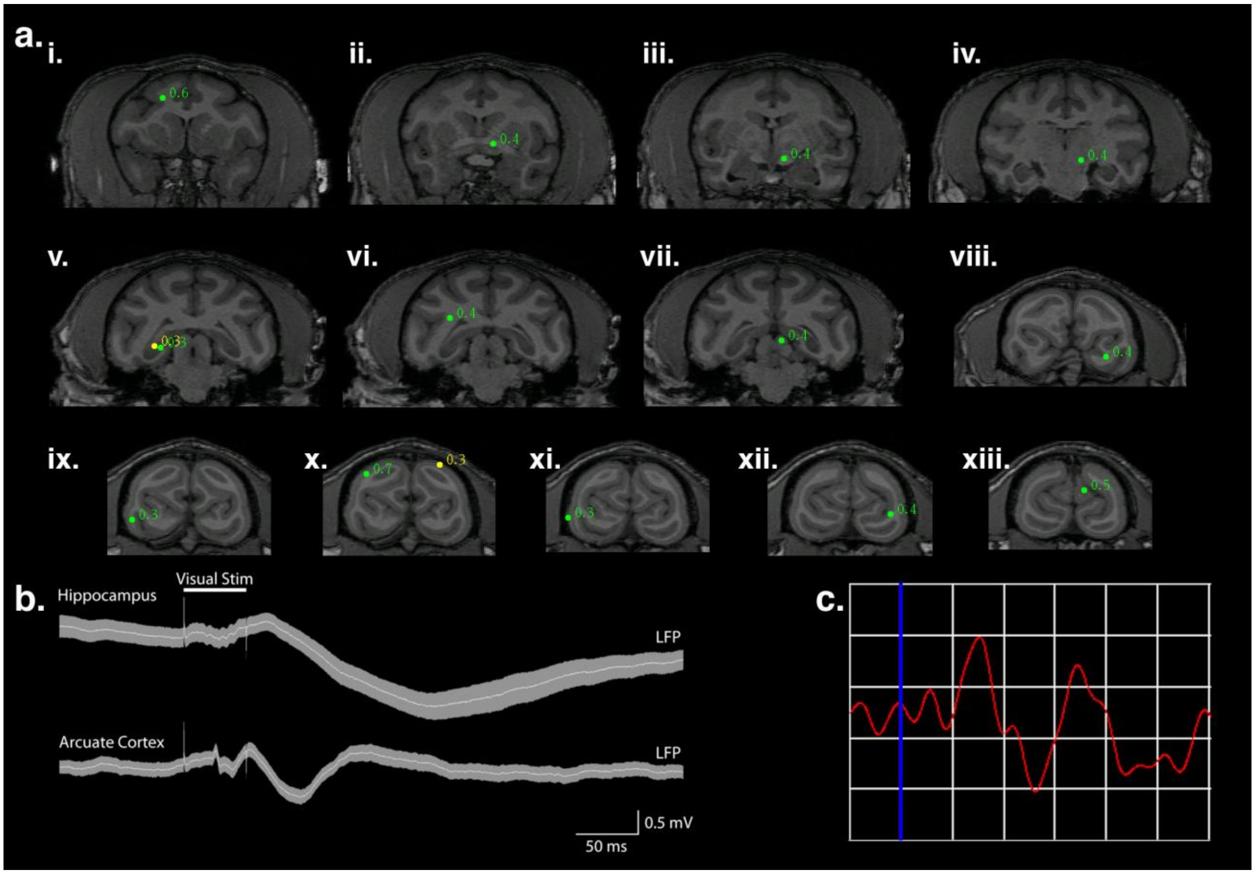

Supplementary Figure 1. Visual stimulation engages related brain regions. a. Identification of functional networks in response to 50 ms light presentations to the left lower visual quadrant of the left eye for NHP M1. SAM analysis of the visual stimulus reveals both peaks in the known visual network as well as additional peaks, including: **i.** right anterior arcuate sulcus, **ii.** left anterior commissure, **iii.** left optic tract, **iv.** left lateral geniculate n. **v.** right posterior hippocampus, **vi.** right posterior white matter tracts, **vii.** left superior colliculus, **viii.** left occipital cortex, V2, **ix.** right primary visual cortex, V1, **x.** bilateral dorsal aspects of V1, **xi.** right primary visual cortex, V1, **xii**. left primary visual cortex, V1, and **xiii.** left occipital cortex. **b.** LFPs recorded from right arcuate cortex and left hippocampus revealed clear event-related potentials in response to visual stimulation. **c.** Average visual evoked field (red trace) derived from virtual electrode for V1 peak in axi. Stimulus onset (0 ms) is given by the blue vertical line. One gray square in the graph = 50 ms on the abscissa and 10 nA-m for the ordinate.

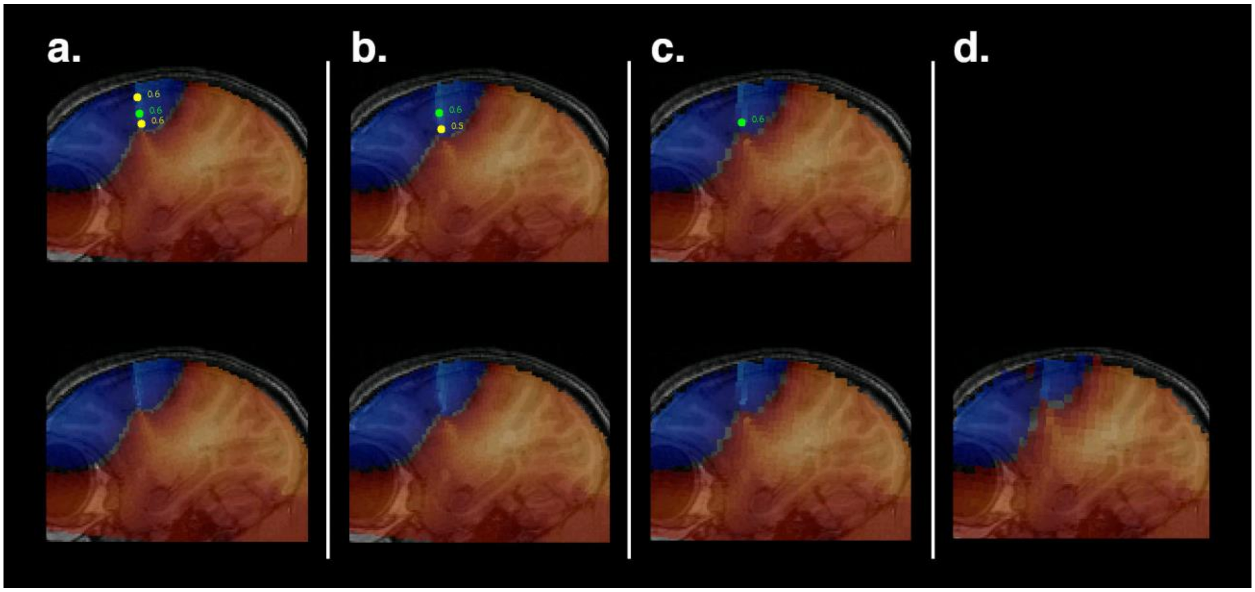

Supplementary Figure 2. Beamforming with different voxel sizes. Sagittal SAM maps of arcuate cortex in NHP M1 with (top row) and without (bottom row) SAM peak t-score values. **a.** At a voxel size of 750 μm three peaks are separable in the map, and a thin, focused band of propagated activity is discernible the map itself around the stimulation site (bottom row). **b.** As the voxel size increases to 1 mm, only two peaks are separable in the SAM map, and the second peak has a reduced t-score of 0.5. The band of activity broadens as well. **c.** At 1.5 mm, only one peak is discernible and is likely the result of two or more true peaks merging. **d.** At 2 mm the SAM map is not sufficiently focused to identify a clear peak in arcuate cortex. The stimuli were 50 ms square light pulses, and the subject and condition are the same as presented in Figure 2a.

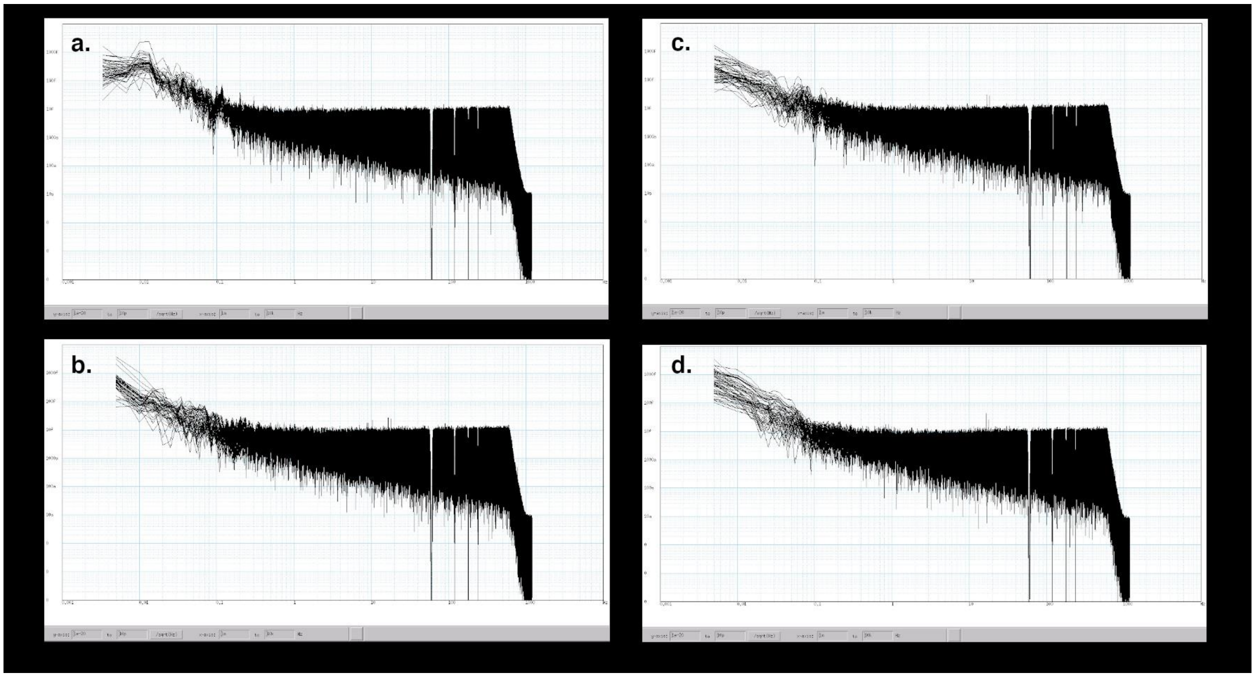

Supplementary Figure 3. Postmortem magnetic fields during optical stimulation are not different from those for an empty room. Presented above are overlaid log-log power spectral densities of all MEG sensors calculated for 0.005-1000 Hz. The spectra are flat from 0.1 Hz to the Nyquist limit of 600 Hz with a noise floor at or below 10 ft/√Hz. A powerline filter was applied for 60 Hz harmonics, but no other preprocessing was applied. **a.** Overlaid power spectra of all MEG channels for an empty room recording. **b.** Overlaid power spectra of all MEG channels obtained from deceased monkey (M1), without optical stimulation. **c.** Overlaid power spectra of all MEG channels obtained from a deceased monkey, with hippocampal optical stimulation. **d.** Overlaid power spectra of all MEG channels obtained from a deceased monkey, with arcuate optical stimulation.

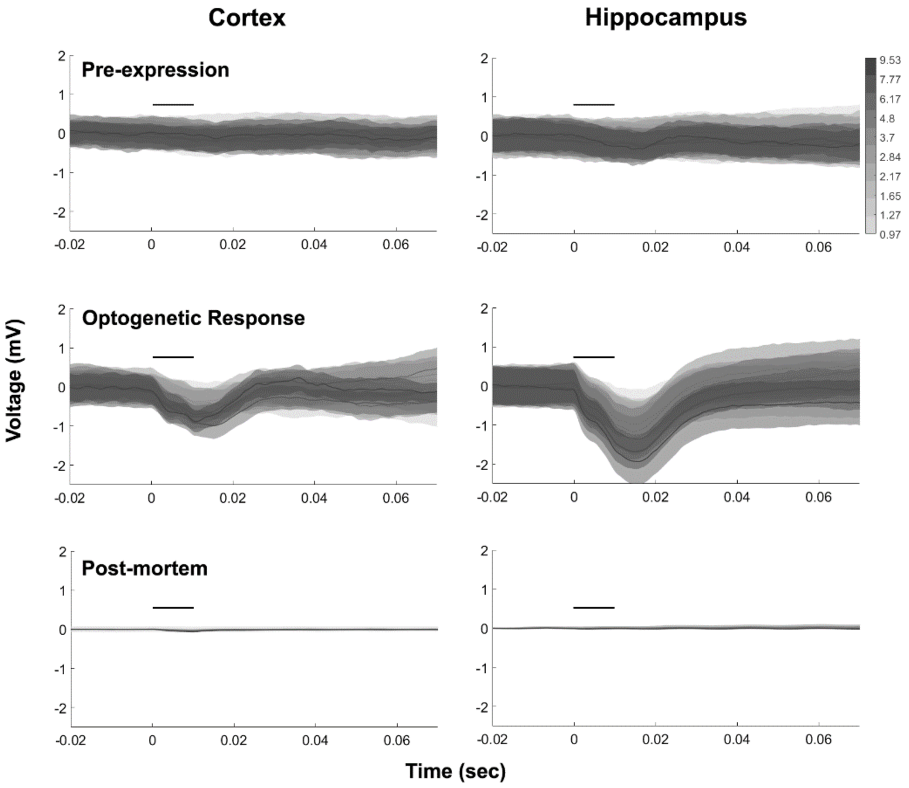

Supplementary Figure 4. Post-mortem stimulation of transduced tissues demonstrates the absence of photovoltaic effect. LFP recordings at three time points in the same subject (M3) demonstrate the appearance of optical response as well as the absence of artifact related to the photovoltaic effect at both transduction sites. Mean evoked LFP with 95% confidence intervals (10 ms pulse, 10 light intensity levels, 20 replicates, 1s ISI). A bandpass filter (1-400 Hz) was applied prior to averaging. Colorbar indicates light intensity in mW. Stimulus and analysis parameters are identical for all recordings.
