# Supplementary material for: MEG source imaging detects optogenetically-induced activity in cortical and subcortical networks
Source: Nat Commun. 2021 Sep 6;12:5259. doi: 10.1038/s41467-021-25481-y (PMC8421372; doi:10.1038/s41467-021-25481-y)
Supplement: Supplementary file 3 — Reporting Summary [file 41467_2021_25481_MOESM3_ESM.pdf]

nature portfolio

Corresponding author(s): Gregory Alberto

Last updated by author(s): Jul 9, 2021

Reporting Summary

Nature Portfolio wishes to improve the reproducibility of the work that we publish. This form provides structure for consistency and transparency in reporting. For further information on Nature Portfolio policies, see our Editorial Policies and the Editorial Policy Checklist.

Statistics

For all statistical analyses, confirm that the following items are present in the figure legend, table legend, main text, or Methods section.

n/a	Confirmed	
☐	☒	The exact sample size (*n*) for each experimental group/condition, given as a discrete number and unit of measurement
☐	☒	A statement on whether measurements were taken from distinct samples or whether the same sample was measured repeatedly
☐	☒	The statistical test(s) used AND whether they are one- or two-sided<br>*Only common tests should be described solely by name; describe more complex techniques in the Methods section.*
☐	☒	A description of all covariates tested
☐	☒	A description of any assumptions or corrections, such as tests of normality and adjustment for multiple comparisons
☐	☒	A full description of the statistical parameters including central tendency (e.g. means) or other basic estimates (e.g. regression coefficient) AND variation (e.g. standard deviation) or associated estimates of uncertainty (e.g. confidence intervals)
☐	☒	For null hypothesis testing, the test statistic (e.g. *F*, *t*, *r*) with confidence intervals, effect sizes, degrees of freedom and *P* value noted<br>*Give P values as exact values whenever suitable.*
☒	☐	For Bayesian analysis, information on the choice of priors and Markov chain Monte Carlo settings
☒	☐	For hierarchical and complex designs, identification of the appropriate level for tests and full reporting of outcomes
☒	☐	Estimates of effect sizes (e.g. Cohen's *d*, Pearson's *r*), indicating how they were calculated

Our web collection on statistics for biologists contains articles on many of the points above.

Software and code

Policy information about availability of computer code

Data collection	MEG data were collected using commercially available software from the manufacturer of the MEG recording hardware. LFP data were collected using DataWave version 9.
Data analysis	MEG data were analyzed using commercially available software from the manufacturer of the MEG system. LFP data were analyzed using custom Matlab scripts available by request and at link provided in manuscript (github.com/neuroptics/optoDR).

For manuscripts utilizing custom algorithms or software that are central to the research but not yet described in published literature, software must be made available to editors and reviewers. We strongly encourage code deposition in a community repository (e.g. GitHub). See the Nature Portfolio guidelines for submitting code & software for further information.

Data

Policy information about availability of data

All manuscripts must include a data availability statement. This statement should provide the following information, where applicable:

- Accession codes, unique identifiers, or web links for publicly available datasets
- A description of any restrictions on data availability
- For clinical datasets or third party data, please ensure that the statement adheres to our policy

Data available from the corresponding author on reasonable request. Data availability partially restricted due to ongoing investigations using the data presented in this manuscript. All data presented in this manuscript will be made available by request.

Field-specific reporting

Please select the one below that is the best fit for your research. If you are not sure, read the appropriate sections before making your selection.

☒ Life sciences ☐ Behavioural & social sciences ☐ Ecological, evolutionary & environmental sciences

For a reference copy of the document with all sections, see nature.com/documents/nr-reporting-summary-flat.pdf

Life sciences study design

All studies must disclose on these points even when the disclosure is negative.

Sample size	All analysis was performed using a within subjects design. The result of optogenetic stimulation was compared to pre-expression baseline recordings. Replicate experiments (n=3) were sufficient to demonstrate generalizability across multiple subjects, but between subject comparisons were not a part of this study design. Sample size was chosen on a balance of available animals while respecting the ethical principle to minimize live animal requirements.
Data exclusions	MEG data were visually inspected for artifact rejection consistent with standard practice. The standard practice is to exclude very high amplitude, very high frequency signals appearing simultaneously across channels. Very high amplitude signals typically saturate the amplifiers and are often identifiable by clipping artifact. Artifact rejection is a pre-established protocol common to all MEG studies.
Replication	Replication was ensured by repeating the preparation in triplicate in three different animals. Each animal was recorded and stimulated in multiple sessions across days to ensure the demonstrated result was robust to preparation and recording environment.
Randomization	All animals were treated with the same preparation and received the same stimulations. Stimulation order was randomized using custom MatLab scripts within experiments to avoid ordering effects (available at above github address).
Blinding	Blinding was not possible as information regarding the optical stimulation is necessary to conduct analysis.

Reporting for specific materials, systems and methods

We require information from authors about some types of materials, experimental systems and methods used in many studies. Here, indicate whether each material, system or method listed is relevant to your study. If you are not sure if a list item applies to your research, read the appropriate section before selecting a response.

Materials & experimental systems

n/a	Involved in the study
☒	☐ Antibodies
☒	☐ Eukaryotic cell lines
☒	☐ Palaeontology and archaeology
☐	☒ Animals and other organisms
☒	☐ Human research participants
☒	☐ Clinical data
☒	☐ Dual use research of concern

Methods

n/a	Involved in the study
☒	☐ ChIP-seq
☒	☐ Flow cytometry
☒	☐ MRI-based neuroimaging

Animals and other organisms

Policy information about studies involving animals; ARRIVE guidelines recommended for reporting animal research

Laboratory animals	Study was repeated in three female vervet monkeys, Chlorocebus aethiops, aged 15-16years old weighing between 4.88-6.75kg.
Wild animals	No wild animals were used.
Field-collected samples	No field collected samples were used
Ethics oversight	All studies were reviewed and approved by the Animal Care and Use Committee of Wake Forest School of Medicine.

Note that full information on the approval of the study protocol must also be provided in the manuscript.
